# Effects of TRPC1’s Lysines on Heteromeric TRPC5-TRPC1 Channel Function

**DOI:** 10.3390/cells13232019

**Published:** 2024-12-06

**Authors:** Isaac S. Demaree, Sanjay Kumar, Kayla Tennessen, Quyen Q. Hoang, Fletcher A. White, Alexander G. Obukhov

**Affiliations:** 1 Department of Anatomy, Cell Biology & Physiology, Indiana University School of Medicine, Indianapolis, IN 46202, USA; idemaree@iu.edu (I.S.D.); sanjaycdri@gmail.com (S.K.); 2 Department of Life Science, School of Earth, Biological, and Environmental Sciences, Central University of South Bihar, Gaya 824236, India; 3 Department of Biochemistry and Molecular Biology, Indiana University School of Medicine, Indianapolis, IN 46202, USA; ktenness@iu.edu (K.T.); qqhoang@iu.edu (Q.Q.H.); 4 Stark Neurosciences Research Institute, Indiana University School of Medicine, Indianapolis, IN 46202, USA; fawhite@iu.edu; 5 Department of Anesthesia, Indiana University School of Medicine, Indianapolis, IN 46202, USA

**Keywords:** TRPC channels, cation channels, electrophysiology, molecular dynamics, TRPC1-TRPC5 heteromers

## Abstract

Background: TRPC5 proteins form plasma membrane cation channels and are expressed in the nervous and cardiovascular systems. TRPC5 activation leads to cell depolarization and increases neuronal excitability, whereas a homologous TRPC1 inhibits TRPC5 function via heteromerization. The mechanism underlying the inhibitory effect of TRPC1 in TRPC5/TRPC1 heteromers remains unknown. Methods: We used electrophysiological techniques to examine the roles of subunit stoichiometry and positively charged luminal residues of TRPC1 on TRPC5/TRPC1 function. We also performed molecular dynamics simulations. Results: We found that increasing the relative amount of TRPC1 in TRPC5/TRPC1 heteromers reduced histamine-induced cation influx through the heteromeric channels. Consistently, histamine-induced cation influx was small in cells co-expressing TRPC5-TRPC1 concatemers and TRPC1, and large in cells co-expressing TRPC5-TRPC1 concatemers and TRPC5. Molecular dynamics simulations revealed that the TRPC1 protein has two positively charged lysine residues that are facing the heteromeric channel pore lumen. Substitution of these lysines with asparagines decreased TRPC1’s inhibitory effect on TRPC5/TRPC1 function, indicating that these lysines may regulate cation influx through TRPC5/TRPC1 heteromers. Additionally, we established that extracellular Mg^2+^ inhibits cation influx through TRPC5/TRPC1, contributing to channel regulation. Conclusions: We revealed that the inhibitory effect of TRPC1 on heteromeric TRPC5/TRPC1 function likely involves luminal lysines of TRPC1.

## 1. Introduction

Transient Receptor Potential Canonical (TRPC) channels are plasma membrane cation channels that allow Na^+^ and Ca^2+^ influx across the cell membrane [1]. There are seven homologous proteins (TRPC1-7) comprising the TRPC subfamily. While there are differences between the members of this group, they share the same overall structure. The structures for TRPC3, TRPC4, TRPC5, and TRPC6 proteins have been solved [2,3,4]. The proteins contain six transmembrane α-helices and long cytosolic N- and C-termini. The S6 transmembrane domain residues line the lumen of the channel pore, and the pore loop forms the selectivity filter in TRPC channels [5]. It was proposed that TRPCs have two gates, namely, the upper gate, which serves as the selectivity filter, and the lower gate that is critical for closing the pore lumen at rest. The TRPC subfamily can be further subdivided into smaller subgroups that exhibit closer amino acid sequence similarities. These are the following groups: (1) TRPC1, (2) TRPC2, (3) TRPC3, TRPC6, and TRPC7, and (4) TRPC4 and TRPC5 [1,6]. The biochemical, biophysical, and structural studies revealed that a functional TRPC channel is composed of four subunits and can be either homo- or heterotetrameric [1,7]. Remarkably, the heteromerization between TRPC1 and TRPC5 proteins occurs frequently in the brain and in the cardiovascular system [8,9,10]. TRPC channels are expressed in virtually all organ systems of the body [1]. These channels have been implicated in many disease states, including hypoxic pulmonary hypertension, cardiac hypertrophy, atherosclerosis, traumatic brain injury-induced endothelial dysfunction, kidney disease, and others [1,6,11,12]. In this study, we focus on the role of TRPC1 in regulating TRPC5 function by heteromerization.

TRPC5 is expressed in the nervous and cardiovascular systems [12,13,14]. The human *TRPC5* gene was mapped to the Xq23 segment on the X-chromosome. This segment is associated with nonsyndromic mental retardation. Remarkably, TRPC5 channels can be activated by many different signals, including store depletion and ligands of G-protein-coupled receptors, such as histamine [15]. Animal studies revealed that TRPC5 may be involved in regulating neurite length and growth cone morphology [16,17], nociception, conditioned fear responses [18,19], and the cerebral ischemia-reperfusion injury-associated death of neurons [20]. Additionally, increased TRPC5 activity has been associated with seizures, epileptogenesis, and excitotoxicity [21]. TRPC5 and heteromeric TRPC5/TRPC1 channels are also expressed in vascular smooth muscle and endothelial cells, and they may contribute to regulating vascular tone [10,22,23,24,25]. Furthermore, elevated levels of TRPC5 expression were found in the monocytes of hypertensive patients [26].

TRPC1 is widely expressed in the nervous and cardiovascular systems [27,28]. For example, the expression of TRPC1 and TRPC5 in the hippocampus has been shown to be important for working memory and learning [29]. As we noted above, TRPC5 does not always work alone and is also capable of forming heterotetramers with TRPC1. This was first noted by the Clapham lab in neurons of the hippocampus [8]. The Clapham group also provided evidence that heteromeric proteins may be formed among TRPC1, TRPC3, TRPC5, and TRPC6 in the mammalian brain [30]. It was noted by them and others that TRPC5/TRPC1 heterotetramers exhibit markedly reduced cation influx at physiological membrane potentials compared to homotetrameric TRPC5 [8], indicating that TRPC1 has an inhibitory effect on TRPC5 function. Consistently, it was recently reported that TRPC1 knockout mice exhibited increased metabotropic G-protein-coupled glutamate receptor (mGluR I) activation-dependent inward currents in hippocampal CA1 neurons compared to those in CA1 hippocampal neurons of wild-type mice [31]. It was demonstrated that heteromeric TRPC5/TRPC1 channels are activated in a similar manner as TRPC5 [32]. Despite all of this research, the underlying mechanisms involved in the inhibitory role of TRPC1 on TRPC5 function remain to be elucidated.

The inhibitory effect of TRPC1 on TRPC5 function can be modeled in the HEK mammalian heterologous expression system. Indeed, the TRPC5/TRPC1 heteromer has a markedly altered current-voltage relationship compared to the homomeric TRPC5 channel. TRPC5 homomers show large inward currents at physiologically negative membrane potentials and an S-shaped current-voltage (I–V) relationship, whereas TRPC5/TRPC1 heteromers exhibit smaller inward currents at negative membrane potentials and an outwardly rectifying I–V relationship, dramatically different from that of homomeric TRPC5 channels [8]. Furthermore, it was demonstrated that heterologously expressed heterotetrameric TRPC5/TRPC1 channels exhibit not only reduced conductance at physiological potentials but also decreased calcium permeability compared to the homomeric TRPC5 channels [33].

As we indicate above, the TRPC1 channel is more likely to play a regulatory role [34], although there are reports indicating that TRPC1 itself may form a functional channel with variable gating mechanisms. It has been suggested that TRPC1 may be retained in the endoplasmic reticulum and fails to localize to the plasma membrane when it is expressed in the absence of TRPC4 or TRPC5 [35]. It is, however, unreasonable to assume this means that TRPC1 alone cannot function as a homotetrameric channel. Studies have shown that TRPC1 is highly expressed in breast cancer and may serve a protective role in limiting the proliferation and migration of breast cancer cells [36]. TRPC1 dysfunction has also been implicated in other types of cancer such as pancreatic, lung, and hepatic cancers, among others [37]. It has also been indicated that the deletion of TRPC1 increases memory loss and cell death related to amyloid-β in subjects with Alzheimer’s Disease [38]. Recent advances in understanding the physiological functions of TRPC1, TRPC5, and TRPC5/TRPC1 heteromers have been fueled by the discoveries of multiple small molecular modulators of TRPC5 and TRPC5/TRPC1 channels [39]. For example, both TRPC5 and TRPC5/TRPC1 can be directly activated by (−)-englerin A and tonantzitlolone, and inhibited by Pico145 and HC-070 [32].

In this study, we focused on the histamine-induced route of activation of TRPC5 and TRPC5/TRPC1 heteromers to determine the effect of stoichiometry on the biophysical properties of TRPC5/TRPC1 heterotetrameric channels compared to TRPC5 homomeric channels. To further this, we employed the TRPC5-TRPC1 concatemers to more accurately control the relative TRPC5/TRPC1 stoichiometry in heteromeric channels. While identifying the mechanisms responsible for the inhibitory effect of TRPC1, we discovered the unique role of two lysine residues in TRPC1 flanking the putative lower gate in the heterotetrameric TRPC5/TRPC1 channel. Finally, we revealed the role of the extracellular Mg^2+^ block of the heterotetrameric channel pore for regulating Na^+^ influx through the TRPC5/TRPC1 heteromeric channel.

## 2. Materials and Methods

### 2.1. Molecular Dynamic Simulation

A homology model of mTRPC1 (residues 538–680) constructed using the cryo-EM structure of mTRPC5 (PDB ID: 6AEI) as template was used as a starting model for the simulation. QwikMD 1.9.3 [40] was used to add hydrogen atoms and prepare the model for simulation. Equilibration of the system at room temperature (300 K) and 1 atmosphere pressure (101.3 kPa) was performed with the NAMD 3.0 program [41], with implicit solvation at a neutral pH and 150 mM NaCl, using the CHARMM36 force field [42]. The 50-nanosecond simulation was carried out at fixed temperature (300 K) and pressure (1 atm) using the Langevin piston method [43]. The results were examined using Coot 0.8.9.1 [44], and the figures were prepared using PyMol 2.4.0 (Schrödinger, LLC, Cambridge, MA, USA).

### 2.2. Molecular Biology

The mouse TRPC5 (accession #NM_009428), mouse TRPC1 (accession # U73625), and mouse H1 histamine receptor (H1R) (accession #D50095) cDNAs were used in this study [7]. All of the TRPC1 mutants were generated using the Lightening QuikChange site-directed mutagenesis kit (Agilent Technologies, Santa Clara, CA, USA) according to the manufacturer’s recommendations. All of the mutations were verified by sequencing. The TRPC5-TRPC1 concatemer was engineered by removing the stop codon in the mouse TRPC5 open reading frame cDNA and cloning it into the TRPC1-pcDNA3 plasmid using the Acc651 and Sac II restriction sites, which resulted in the mTRPC5-mTRPC1-pcDNA3 construct with a 28-amino-acid linker of GSNPRHQGQFCRYPAQWRPLESRGPAAT connecting the C-terminus of mTRPC5 and the N-terminus of mTRPC1.

### 2.3. HEK Cell Culture and Transfection

Eagle’s minimum essential medium supplemented with 10% fetal bovine serum was used to culture the HEK cells (293T/17, CRL-11268, American Type Culture Collection, Manassas, VA, USA). Lipofectamine 3000 reagent (Invitrogen, Waltham, MA, USA) was used in accordance with the manufacturer’s instructions to transfect the HEK cells. The transfection mixture contained 4 μg of each channel cDNA and 0.25 μg of the histamine H1 receptor. For each batch of transfected cells, 24–48 h of culturing time was allowed before the cells were taken for the electrophysiological experiments. The cells were also transfected with variable amounts of indicated channel subunit cDNAs to adjust the relative expression of TRPC1 versus TRPC5. Additionally, 2 μg of TRPC5-TRPC1 tandem and either 2 μg of TRPC1 cDNA or 2 μg of TRPC5 cDNA were co-expressed together to obtain cells expressing mostly TRPC1 with a minor contribution of TRPC5, or mostly TRPC5 with a minor contribution of TRPC1.

### 2.4. Cell-Surface Protein Biotinylation

Cells were transfected in 35-mm Petri dishes for 48 h in a humidity-controlled CO_2_ incubator using Lipofectamine 3000 (Invitrogen, Waltham, MA, USA). The old culture medium was removed and the cells were washed three times with 1 mL of ice-cold PBS. An amount of 0.5 mL of 50 μM biotin-X-NHS (biotin N-hydroxysuccinimide-sulfo ester; Thermo Fisher Scientific) biotinylating solution in PBS was added to the cells for 30 min. Incubation was carried out at 4 °C. Cells were again washed 3 times with 1 mL of ice-cold PBS, which was supplemented with 100 mM glycine to quench the excess of biotin-X-NHS reagent. After that, the cells were again washed 3 times with 1 mL of PBS.

### 2.5. Western Blotting

Biotinylated cells were lysed by adding 200 μL of the radioimmune precipitation assay buffer (Thermo Fisher Scientific, Waltham, MA, USA). The buffer was supplemented with the halt proteinase inhibitors (Thermo Fisher Scientific, Waltham, MA, USA), phenylmethylsulfonyl fluoride (Thermo Fisher Scientific), and 2% Triton X-100. The lysates were cleared by spinning down at 24,000 × *g* at 4 °C and then mixed with 100 μL of pre-absorbed avidin-agarose bead suspension (Thermo Fisher Scientific, Waltham, MA, USA) as recommended by the manufacturer. After incubation for 30 min at 4 °C, the mixture was spun down and washed 3 times with the complete lysis buffer (200 μL). Proteins of the lysates were separated by 10.5% SDS-polyacrylamide gel electrophoresis (4% stacking gel) and wet-transferred onto a polyvinylidene difluoride membrane (PVDF) (Bio-Rad, Hercules, CA, USA). Membranes were blocked with StartingBlock blocking buffer (Thermo Fisher Scientific, Waltham, MA, USA) for 1 h at room temperature and then incubated overnight at 4 °C with the primary monoclonal TRPC1 antibody (a gift from Dr. Tsiokas; diluted 1:1000) in StartingBlock buffer. Membranes were washed 3 times and then incubated with an anti-mouse secondary antibody conjugated with the horseradish peroxidase (Pierce, Rockford, IL, USA; diluted 1:20,000) for 1 h at room temperature. The SuperSignal West Pico enhanced chemiluminescence Kit (Pierce, Waltham, MA, USA) was used to detect HRP conjugates, as recommended by the manufacturer.

### 2.6. Patch-Clamp Electrophysiology

The whole-cell patch-clamp method was used to measure the ion current across the cell membrane. An Axopatch 200B amplifier and Digidata 1550A digitizer (Molecular Devices, San Jose, CA, USA) were employed to record all of the currents. The sampling rate was 1 kHz, and the currents were filtered at 3 kHz. The pCLAMP 10 software was used for acquisition control and data analyses. The cells were voltage-clamped at a holding potential of −60 mV; 300 ms voltage ramps were applied repeatedly from −100 mV to 100 mV every 2 s. TRPC currents were activated by bathing the cells in 100 μM of histamine extracellular solution. Current-voltage relationships where the leak current was greater than 100 pA and/or the excess resistance was >10 megaohms were excluded from the analysis. As an important parameter of the TRPC characterization, the relative permeability ratios were calculated using the following Equation (1):

(1) $$\frac{P_{Ca}}{P_{Na}}=\frac{[Na^{+}{]}_{o}}{4\times [Ca^{2+}{]}_{0}}\times exp\left(\frac{F\times \left(E_{Ca^{2+}}-E_{Na^{+}}\right)}{RT}\times \left(1+\exp\left(\frac{F\times E_{Ca^{2+}}}{RT}\right)\right)\right)$$

where ENa^+^ and ECa^2+^ are the reversal potentials of the TRPC currents recorded in 150 mM NaCl and 10 mM Ca^2+^-NMDG^+^, respectively; R, T, and F are the absolute gas constant, the temperature in Kelvin, and the Faraday constant, respectively. Current recordings were normalized by cell capacitance to give a measure of the current density. All of the recordings were taken at room temperature (22–25 °C).

### 2.7. Solutions

The standard extracellular solution contained the following (ES-1Ca, in mM): 145 NaCl, 1 CaCl_2_, 1 MgCl_2_, 2.5 KCl, 10 HEPES, and 5.5 glucose (pH adjusted to 7.2 with Trizma base). The pipette solution contained the following (in mM): 10 HEPES, 0.5 EGTA, 140 CsMeSO_3_, 10 CsCl, and 2 MgCl_2_. The NMDG^+^-0Ca solution contained the following (in mM): 150 NMDG, 10 HEPES, 5.5 glucose, and 0.5 EGTA (pH adjusted to 7.2 with Trizma base). The NaCl-EGTA solution contained the following (in mM): 150 NaCl, 10 HEPES, 5.5 glucose, and 0.5 EGTA (pH adjusted to 7.2 with Trizma base). The NMDG^+^-10Ca solution contained the following (in mM): 135 NMDG, 10 HEPES, 10 CaCl_2_, and 5.5 glucose (pH adjusted with Trizma base). The NaCl-1Mg solution contained the following (in mM): 150 NaCl, 10 HEPES, 5.5 glucose, 0.5 EGTA, and 1 MgCl_2_ (pH adjusted to 7.2 with Trizma base). The osmolarity of all solutions was determined and adjusted to 295–305 mOsm by adding mannitol powder.

### 2.8. Drugs

Histamine, glucose, Trizma base, NMDG, HEPES, and other inorganic salts used in the experimental solutions were purchased from Sigma-Aldrich (St. Louis, MO, USA) and had the highest purity.

### 2.9. Statistical Methods

Sigma Plot 12.5 (Inpixon, Palo Alto, CA, USA) was used for the statistical analyses. The data distribution normality was evaluated using the Shapiro-Wilk test. To compare two data sets with normally distributed populations and equal variances, the unpaired t test was used. In the cases of the analysis of two non-normally distributed groups, the Mann-Whitney Rank Sum Test was used to determine whether there was a statistically significant difference between the two data sets. The one-way ANOVA test, followed by an indicated post hoc comparison test, was used to determine whether there was a statistically significant difference between multiple groups presenting normally distributed populations with equal variances, whereas, for non-normally distributed populations with different variances, the one-way ANOVA on rank test, followed by a post hoc comparison test, was used to determine whether there was a statistically significant difference. The data sets were considered significantly different if the *p* value was < 0.05. The data are presented as the means ± SEM.

## 3. Results

### 3.1. The Role of Subunit Stoichiometry on TRPC5/TRPC1 Heteromer Function

**Figure 1:**
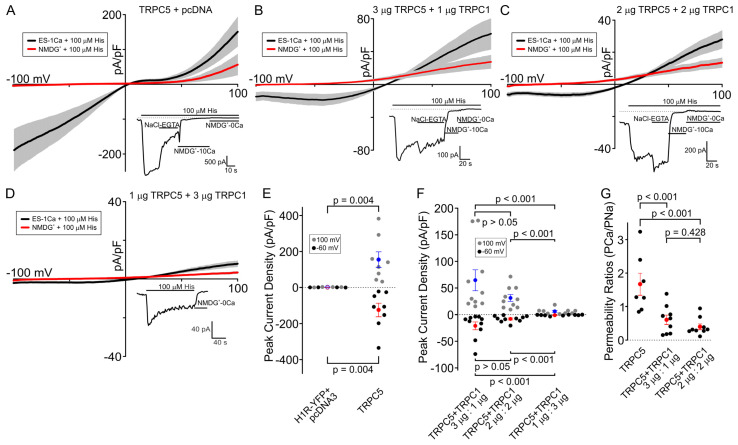
Effect of the TRPC5/TRPC1 heteromeric channel subunit composition on its function. Comparison of histamine-induced current amplitudes in HEK cells expressing either TRPC5 homomers or TRPC5/TRPC1 heteromers with varying ratios of TRPC5 and TRPC1. The cells were transfected with the indicated amounts of channel subunit cDNAs to adjust the relative expression of TRPC1 versus TRPC5 in resulting TRPC5/TRPC1 heteromeric channels. (**A**–**D**) Black curves represent the average current in the presence of ES-1Ca + 100 µM histamine. Red curves show the average current under NMDG^+^-0Ca + 100 µM histamine. The insets show the time course of currents activated by histamine in the presence of extracellular solutions, as indicated by the horizontal bars. Dotted lines indicate the position of zero current in the inset graphs. (**E**) Comparison of the peak current density activated by histamine in HEK cells expressing either H1R alone or both H1R and TRPC5 at both −60 mV (black dots) and 100 mV (gray dots). The red (−60 mV) and blue (100 mV) dots represent the mean of each set, and the error bars show the standard error of the mean (SEM). The Mann–Whitney Rank Sum Test was used to analyze the data. (**F**) Comparison of histamine-activated currents at −60 mV (gray dots) and 100 mV (black dots) for varying ratios of TRPC5/TRPC1 heteromeric channels. The red (−60 mV) and blue (100 mV) dots represent the mean for each set, and the error bars show the SEM. The Kruskal-Wallis one-way analysis of variance on ranks with Dunn’s post hoc test was used to analyze the data. (**G**) Comparison of the permeability ratios of inward currents through TRPC5 homomers and two ratios of TRPC5/TRPC1 heteromers. A one-way analysis of variance with the Student-Newman-Keuls post hoc test was used to analyze the data. The shown data points represent independent biological replicates.

It has been reported by previous studies that the biophysical properties of TRPC5/TRPC1 heteromers differ significantly from those of homomeric TRPC5 channels [8]. Thus, it is apparent that TRPC5/TRPC1 heteromeric channel formation may attenuate the effect of activated homomeric TRPC5 channel-mediated depolarization on neural excitability, which may explain its putative role in epileptogenesis. We used the patch-clamp whole-cell approach and measured the currents through the homomeric TRPC5 channel, and compared them with the currents through the heteromeric TRPC5/TRPC1 channels co-expressed with the H1-histamine receptor (H1R) in the HEK cells. HEK cells expressing TRPC5 and H1R in the absence of TRPC1 exhibited a large inward current at −60 mV (−122.27 ± 37.51 pA/pF) and outward currents at 100 mV (154.79 ± 43.53 pA/pF) when the cells were stimulated with a saturating concentration of histamine of 100 μM (Figure 1A). As expected, when TRPC1 was co-expressed with TRPC5 with a cDNA ratio of 1:1 (2 μg TRPC5 cDNA + 2 μg TRPC1 cDNA) along with H1R (0.25 μg cDNA) in the HEK cells, the histamine-induced inward currents had significantly reduced amplitudes at −60 mV (−6.41 ± 1.37 pA/pF, *p* < 0.001) as compared to the HEK cells expressing only TRPC5 and H1R (Figure 1C). Notably, instead of the typical S-shaped I–V relationship of homomeric TRPC5 (Figure 1A), we observed an outwardly rectifying I–V relationship in the HEK cells expressing both TRPC5 and TRPC1 along with H1R (Figure 1B,C), as reported before by others and by us [7,8]. The amplitude of the histamine-induced currents were greater in the HEK-TRPC5 cells compared to the HEK cells co-expressing variable cDNA ratios of TRPC1 and TRPC5 (Figure 1E,F). The calculated permeability ratios for Ca^2+^ over Na^+^ significantly decreased from 1.7 ± 0.3 for TRPC5 to 0.4 ± 0.3 (*p* < 0.001) for TRPC5/TRPC1 channels with a TRPC5:TRPC1 ratio of 1:1 (Figure 1G), which is consistent with the previous report [33] and supports the hypothesis that TRPC1 can inhibit TRPC5-mediated Ca^2+^ influx by forming TRPC5/TRPC1 heteromers in cells natively expressing both of these channels.

We next hypothesized that the stoichiometry of TRPC5/TRPC1 heteromers can play a role in regulating inward currents. To test this, we performed experiments varying the ratios of the TRPC1 and TRPC5 cDNA amounts during transfection to modulate the TRPC5/TRPC1 heteromeric channel stoichiometry. We found that the histamine-induced inward cation currents through TRPC5/TRPC1 heteromers were large (−18.03 ± 6.82 pA/pF) at −60 mV when the HEK cells were transfected with a cDNA ratio of 3 μg:1 μg (TRPC5:TRPC1) and much smaller (−1.81 ± 0.53 pA/pF, *p* < 0.001; Figure 1F) when the HEK cells were transfected with a cDNA ratio of 1 μg:3 μg (TRPC5:TRPC1). The amplitudes of the inward currents through the heteromeric TRPC5/TRPC1 in the cells transfected with a cDNA ratio of 1 μg:3 μg were also significantly smaller than those observed in the cells expressing the heteromeric TRPC5/TRPC1 channels after cell transfection with a ratio of 2 μg:2 μg (Figure 1C,D,F). However, there was no significant difference between the amplitudes of the inward currents through the heteromeric 3 μg:1 μg TRPC5/TRPC1 channels and the 2 μg:2 μg TRPC5:TRPC1 heteromeric currents recorded at −60 mV (Figure 1F).

### 3.2. Heteromeric Channels Composed of the TRPC5-TRPC1 Concatemer and TRPC5 Exhibit Larger Inward Currents Compared to TRPC5-TRPC1 Concatemer/TRPC1 Heteromers

**Figure 2:**
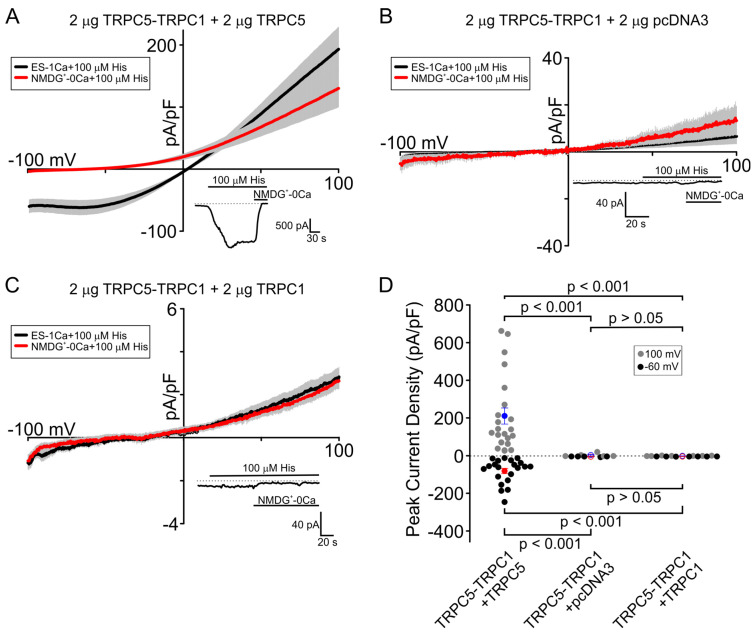
Effect of different ratios of TRPC5 and TRPC1 on the function of the heteromeric TRPC5/TRPC1 channel containing the TRPC5-TRPC1 concatemer. The cells were transfected with the indicated amounts of channel subunit cDNAs. (**A**–**C**) Black curves represent the average current in the presence of ES-1Ca + 100 µM histamine. Red curves show the average current under NMDG^+^-0Ca + 100 µM histamine. The insets show the time course for the inward current activated by histamine under extracellular solutions, as indicated by the horizontal bars. Dotted lines indicate the position of zero current level in the inset graphs. (**D**) Comparison of the peak current density activated by histamine in the HEK cells expressing different TRPC5-TRPC1 concatemer combinations at both −60 mV (black dots) and 100 mV (gray dots). The red (−60 mV) and blue (100 mV) dots represent the mean of each set of data, and the error bars show the standard error of the mean. The Kruskal-Wallis one-way analysis of variance on ranks with the Dunn’s post hoc test was used to analyze the data. The shown data points represent independent biological replicates.

Another established method of studying the heteromeric TRPC5/TRPC1 channels involves engineering a TRPC5-TRPC1 concatemer in which the TRPC5 and TRPC1 subunits are covalently linked. We found that HEK cells co-expressing TRPC5-TRPC1 concatemers and TRPC1 subunits exhibited inward currents that were very small (−0.29 ± 0.11 pA/pF) at −60 mV compared to those observed in HEK cells co-expressing the TRPC5-TRPC1 concatemer and TRPC5 (−61.22 ± 11.51 pA/pF, −60 mV, *p* < 0.001; Figure 2D). The TRPC5-TRPC1-concatemer-alone group also showed little currents (−0.61 ± 0.58 pA/pF, −60 mV; Figure 2B). These data indicate that the presence of only one TRPC1 subunit in the heteroterameric TRPC5/TRPC1 channels may be sufficient to dramatically reduce cation influx through the channel compared to that through the homomeric TRPC5 channel (−122.27 ± 37.51 pA/pF at −60 mV) and to alter the heteromeric channel’s current-voltage relationship shape.

### 3.3. The Role of Positively Charged Lysines Lining the Pore Lumen of the TRPC5/TRPC1 Heteromeric Channel

The presented evidence thus far indicates that increasing the relative number of TRPC1 subunits in the heteromeric TRPC5/TRPC1 channel is associated with a smaller inward current amplitude. These findings further support the hypothesis that TRPC1 exhibits an inhibitory effect on TRPC5 function. We next set out to identify the molecular determinants contributing to this phenomenon.

**Figure 3:**
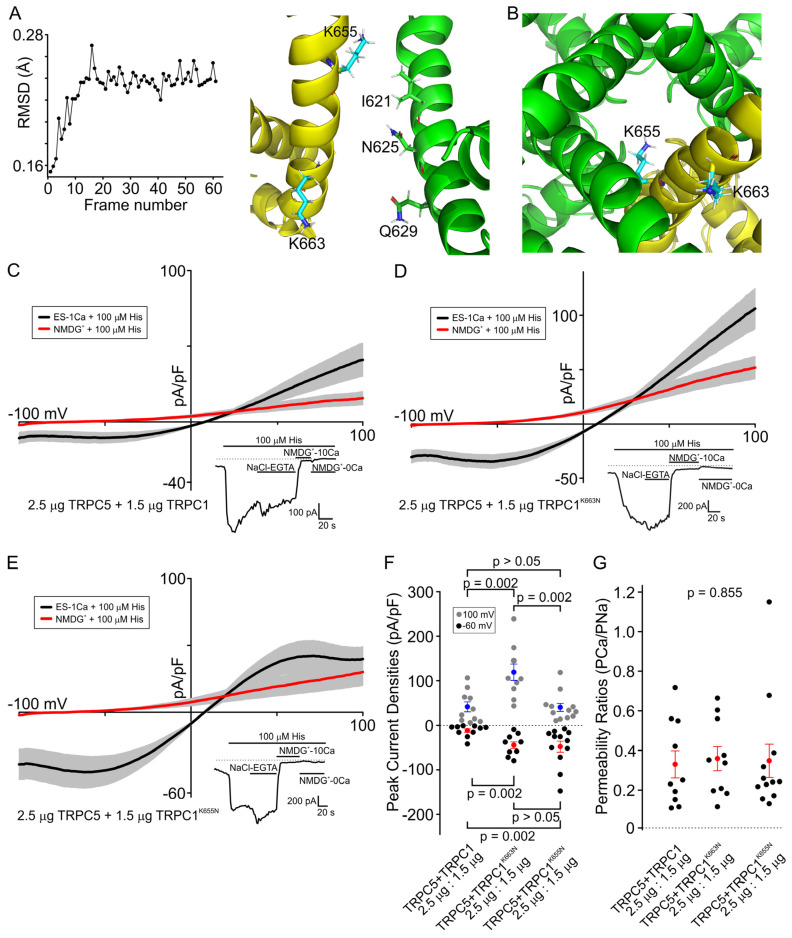
The role of TRPC1’s lysines in TRPC5/TRPC1 heteromeric channel function. The cells were transfected with the indicated amounts of channel subunit cDNAs. (**A**) *Left*, The RMDS plot (left) demonstrates that the simulation system had reached equilibrium before the snapshot (right) was selected from the MD trajectory. *Right*, Molecular dynamics simulations predict that the K655 and K663 residues of TRPC1 likely face the lumen of TRPC5/TRPC1 heteromeric channels. (**B**) A magnified view of the TRPC5/TRPC1 pore from the cytoplasmic side. (**C**–**G**) Comparison of the biophysical properties of the TRPC5/TRPC1 heteromeric channels containing either wild-type TRPC1, TRPC1^K663N^, or TRPC1^K655N^. (**C**–**E**) Black curves represent the average current in the presence of ES-1Ca + 100 µM histamine. Red curves show the average current under NMDG^+^-0Ca + 100 µM histamine. The insets show the time course of the current activated by histamine (100 µM) in the presence of extracellular solutions, as indicated by the horizontal bars. Dotted lines indicate the position of zero current in the inset graphs. (**F**) Comparison of the peak current density activated by histamine in the HEK cells expressing TRPC5 and either WT TRPC1, TRPC1^K663N^, or TRPC1^K655N^ at both −60 mV (black dots) and 100 mV (gray dots). The red (−60 mV) and blue (100 mV) dots represent the mean of each data set, and the error bars show the SEM. The Kruskal-Wallis one-way analysis of variance on ranks with Dunn’s post hoc test was used to analyze the data. (**G**) Comparison of the permeability ratios of the currents recorded in the HEK cells transfected with TRPC5 and WT TRPC1, TRPC1^K663N^, or TRPC1^K655N^ for each experiment (black dots). The red dots represent the mean of each data set, and the error bars show the standard error of the mean. The Kruskal-Wallis one-way analysis of variance on ranks was used to analyze the data. The shown data points represent independent biological replicates.

We started by building an atomic model of a (TRPC5)_3_TRPC1 heteromeric channel by using homology modeling followed by molecular dynamics simulations. Analyzing the resulting model, we noticed two positively charged lysine residues in the TRPC1’s S6 domain, which may be lining the pore lumen of the heteromeric channel pore, and thus could influence the conduction of cations through the lumen of the channel (Figure 3A,B). We also noticed that this pair of lysine residues may flank the putative lower gate of the heteromeric TRPC5/TRPC1 channel, although K663 is situated at a longer distance. In contrast, TRPC5 lacks any positively charged residues in its S6 transmembrane domain, and the homologous positions to the pair of lysines of the TRPC1’s S6 transmembrane domain are occupied by two polar asparagine residues in TRPC5. We note that the MD simulation does not include a lipid bilayer; thus, its use is limited to residues in the aqueous lumen of the channel, which do not interact with the membrane.

Therefore, we next sought to define the role of these lysines of TRPC1. We first mutated the lower lysine to asparagine (TRPC1^K663N^), which is situated below the lower gate of the TRPC5/TRPC1 heteromeric pore. We expressed the TRPC5 cDNA and TRPC1 cDNA at a ratio of 2:1 for both the WT and mutated TRPC1 channel in the HEK cells, and compared the current amplitudes of the WT TRPC5/TRPC1 heteromers (−10.51 ± 3.75 pA/pF) to those of the mutated TRPC5/TRPC1^K663N^ heteromers (Figure 3C,D). We found that the K663N mutant exhibited significantly larger currents at −60 mV (−34.34 ± 6.42 pA/pF, *p* = 0.002). This suggests that lys663 may contribute to regulating the cation flow through the pore of the heteromeric TRPC5/TRPC1 channel.

We next mutated the second lysine located above the putative lower gate of the TRPC1 channel to asparagine (TRPC1^K655N^). We found significantly greater currents in the HEK cells expressing TRPC5/TRPC1^K655N^ heteromeric channels (−44.40 ± 11.89 pA/pF) compared to those expressing the wild-type TRPC5/TRPC1, but not TRPC5/TRPC1^K663N^ (Figure 3C–F). Thus, lys655 apparently plays a similar role to lys663, regulating the cation flow through the heteromeric channel. Remarkably, the TRPC5/TRPC1^K655N^ I–V curve showed a plateaued outward current at higher positive membrane potentials, while the wild-type channels showed increasing currents with rising positive holding potentials (Figure 3C,E). We also determined the permeability ratios for Na^+^ and Ca^2+^, and found that the TRPC5/TRPC1^K655N^ and TRPC5/TRPC1^K663N^ heteromers exhibited similar values to those observed for the WT TRPC5/TRPC1 (Figure 3G).

### 3.4. Extracellular Mg^2+^ Reduces Inward Currents Through the Heteromeric TRPC5/TRPC1 Channel

**Figure 4:**
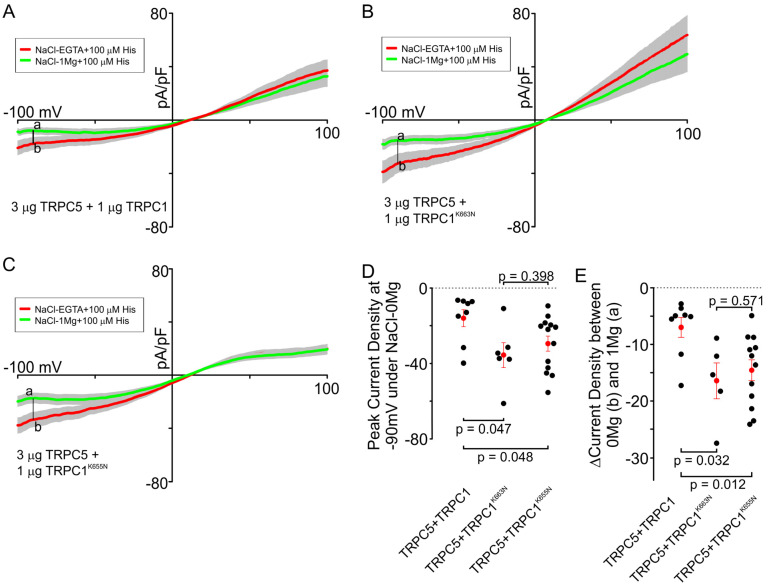
Effect of Mg^2+^ removal on the current-voltage relationships of wild-type TRPC5/TRPC1 and relevant mutants. The cells were transfected with the indicated amounts of channel subunit cDNAs. (**A**–**C**) Red curves represent the average current in the presence of NaCl-EGTA-0Mg + 100 µM histamine. Green curves show the average current under NaCl-EGTA-1Mg + 100 µM histamine. (**D**) Comparison of the peak current densities of TRPC5/TRPC1, TRPC5/TRPC1^K663N^, or TRPC5/TRPC1^K655N^ transfection groups at −90 mV in the absence of Mg^2+^. Black dots represent individual experiments. Red dots represent the mean value. The one-way analysis of variance with the Student–Newman–Keuls post hoc test was used to analyzed the data. (**E**) Comparison of the peak current density in the presence and absence of Mg^2+^ at −90 mV. Each black dot shows the difference between point “b” and point “a” for each experiment. Red dots represent the mean. The one-way analysis of variance with the Student–Newman–Keuls post hoc test was used to analyze the data. Error bars show the standard error of mean. The shown data points represent independent biological replicates.

While determining the permeability ratios, we noticed that the removal of Mg^2+^ from the extracellular solution led to increased inward current amplitudes and a change in the current-voltage relationship for the heteromeric TRPC5/TRPC1 channel. The native TRPC5/TRPC1 current-voltage relationship (TRPC5:TRPC1—3:1) had a maximal inward current amplitude peaking at around −45 mV and showed a decreased current with further hyperpolarization. In the absence of Mg^2+^, the inward current through TRPC5/TRPC1 monotonically increased with hyperpolarization. Therefore, we next investigated the role of Mg^2+^ in modulating cation influx through the TRPC5/TRPC1 heteromeric channels. We found that histamine-activated inward currents through heteromeric TRPC5/TRPC1 channels in a Mg^2+^-free extracellular solution were smaller in WT TRPC5/TRPC1 (−17.74 ± 4.69 pA/pF at −90 mV; 3:1 ratio) compared to both TRPC5/TRPC1^K663N^ and TRPC5/TRPC1^K655N^ (−32.43 ± 7.06 pA/pF, *p* = 0.047, and −33.40 ± 5.59 pA/pF, *p* = 0.048, respectively; 3:1 ratio, Figure 4). When the Mg^2+^-free solution was replaced with 1 mM Mg^2+^ solution, the original shape of the current-voltage relationship recovered, indicating that these changes are reversible. Figure 4E shows the quantification of the data on the removal of Mg^2+^ from the extracellular solution. We observed that the Mg^2+^ block at −90 mV was significantly greater (*p* < 0.05) in both mutants, TRPC5/TRPC1^K655N^ and TRPC5/TRPC1^K663N^, compared to the WT TRPC5/TRPC1. The a–b values (Figure 4A–C) amounted to −6.99 ± 1.75 pA/pF in the WT TRPC5/TRPC1, −14.56 ± 1.83 pA/pF in the TRPC5/TRPC1^K655N^ mutant, and −16.43 ± 3.17 pA in the TRPC5/TRPC1^K663N^ mutant (Figure 4E). Notably, there was no effect of Mg^2+^ removal on the outward currents, likely indicating that the Mg^2+^ effect was voltage-dependent. This is similar to the behavior seen in the glutamate receptor NMDA receptor channels found on the excitatory synapses in the central nervous system. 

### 3.5. Heteromers Containing the Double Mutant of TRPC1^K655N,K663N^ Exhibit Similar Inward Current Amplitudes Compared to TRPC5/TRPC1^K655N^ and TRPC5/ TRPC1^K663N^ Heteromers

**Figure 5:**
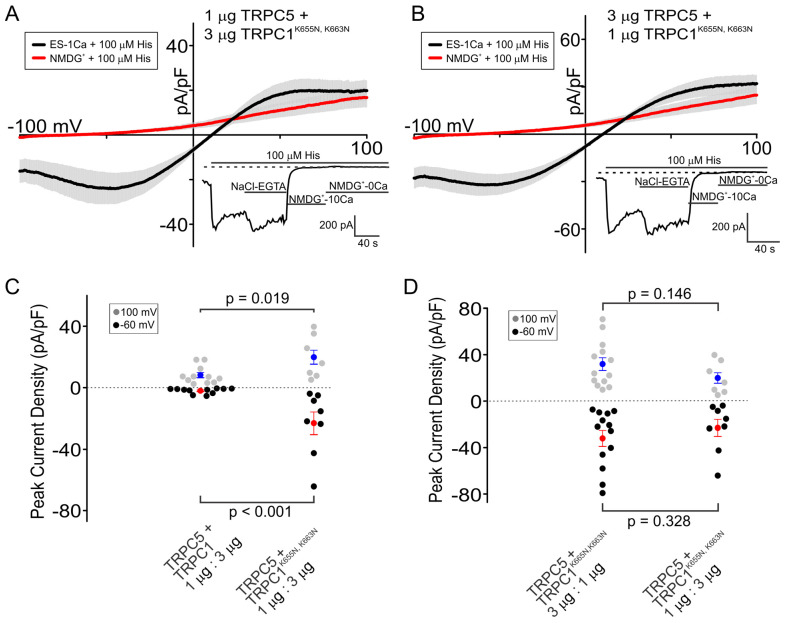
Effect of the double-mutation K663N and K655N on the TRPC5/TRPC1 heteromeric channel function in the HEK cells transfected with variable cDNA ratios. The cells were transfected with the indicated amounts of channel subunit cDNAs. (**A**,**B**) Current-voltage relationships of the TRPC5/TRPC1^K655N,K663N^ channels recorded during voltage ramps from −100 mV to 100 mV. Black curves represent the average current under ES-1Ca + 100 µM histamine. Red curves show the average current under NMDG^+^-0Ca + 100 µM histamine. The insets show a time course showing the development of the current activated by histamine (100 µM) extracellular solutions, as indicated by the horizontal bars. Dotted lines indicate the position of zero current in the inset graphs. (**C**) Comparison of the peak current densities for the 1:3 WT and double-mutant channels at both −60 mV (black dots) and 100 mV (gray dots). The red (−60 mV) and blue (100 mV) dots represent the mean of each data set, and the error bars show the standard error of mean. (**D**) Comparison of the difference in the peak current densities for the 1:3 and 3:1 double-mutant channels at both −60mV (black dots) and 100 mV (gray dots). The red (−60 mV) and blue (100 mV) dots represent the mean of each data set, and the error bars show the standard error of the mean. The Mann-Whitney Rank Sum Test was used to analyze the data. The shown data points represent independent biological replicates.

We next investigated whether the replacement of both K663 and K655 lysines with asparagines would lead to an additive increase in the amplitude of the inward currents through the heteromeric channel. We found that the HEK cells co-expressing the TRPC5 and TRPC1^K663N,K655N^ double mutant at a ratio of 1:3 carried significantly greater inward currents than the wild-type TRPC5/TRPC1 channel with the same stoichiometry (Figure 5). Figure 5A,B show that the shape of the current-voltage relationship was very similar to that of the TRPC5/TRPC1^K655N^ mutant (Figure 3E), indicating that the substitution of the lys655 has a dominant effect on the outward shoulder of the I–V relationship. Notably, the HEK cells expressing the double TRPC5/TRPC1^K663N,K655N^ mutant in a ratio of 1:3 and 3:1 exhibited similar amplitudes of histamine-induced inward currents (−23.15 ± 7.36 pA/pF and −32.14 ± 6.88 pA/pF, respectively; *p* = 0.328, Figure 5D) at −60 mV. These currents were significantly greater (*p* < 0.001) than those observed in the HEK cells expressing WT TRPC5/TRPC1 with 1:3 and 3:1 ratios (−1.13 ± 0.36 pA/pF and −18.03 ± 6.82 pA/pF, respectively; Figure 1B,D and Figure 3C). This indicates that the substitution of both positively charged lysine residues with asparagine improves the ability of the heteromeric channel to conduct cation currents. However, we found that the HEK cells expressing the double TRPC5/TRPC1^K663N,K655N^ mutant exhibited histamine-induced inward currents that were not significantly different from those observed in the HEK cells expressing the TRPC5/TRPC1^K655N^ mutant or the TRPC5/TRPC1^K663N^ mutant alone. This suggests that both positive lysine residues, K663 and K655, contribute to regulating cation influx through the heteromeric TRPC5/TRPC1 channel, though not in an additive fashion.

## 4. Discussion

In this study, we identified two specific lysine residues, K663 and K655, flanking the lower gate of the TRPC5/TRPC1 heteromeric channel that regulate cation influx through the channel at physiological potentials. We and others have demonstrated that TRPC1 is a negative regulator of TRPC5 activity (for a review, see [34]). Consistently, we found that increasing the relative amount of TRPC1 cDNA compared to that of TRPC5 cDNA during transfection to generate a TRPC5/TRPC1 heteromeric channel with a relative stoichiometry of 1:3 decreases the ability of the heteromer to conduct inward cation currents, whereas cells expressing greater levels of TRPC5 compared to that of TRPC1 to generate heteromeric TRPC5/TRPC1 channels with a relative stoichiometry of 3:1 exhibited increased inward cation currents. Thus, an alteration in heteromeric channel stoichiometry due to the differential expression of *TRPC5* and *TRPC1* genes may be an earlier unrecognized endogenous pathway regulating TRPC5 function, and receptor-induced depolarization and excitability in TRPC5-expressing neurons.

TRPC5 has been associated with a number of physiological processes and pathophysiological conditions. TRPC5 channels are involved in growth cone guidance, neurite outgrowth, and increased neuronal excitability, including provoked seizures [16,17]. TRPC5 also appears to be involved in amygdala function, fear conditioning, and learning [18,19], as well as oxidative injury in the settings of acute brain injury and ischemic reperfusion injury [20,45].

TRPC1 is another TRPC protein found throughout the body [27] and functions as a negative regulatory subunit of TRPC4 and TRPC5, or perhaps as a fully functional cation channel in some cell types. It is unclear whether native functional TRPC1 channels are homomeric. While forming heteromeric channels with TRPC5, TRPC1 is known to decrease the inward currents through the TRPC5/TRPC1 heterotetramers at physiological potentials. However, the underlying mechanism is not fully understood.

In this study, we first determined the effect of heteromeric channel stoichiometry alteration on the channel ability to conduct cation influx at negative physiological potentials. We showed that, as the relative expression level of TRPC1 increased compared to that of TRPC5 in the HEK cells, the cation influx through the heteromeric channel decreased. We found that calcium permeability, determined as the permeability ratio of PCa/PNa, was similarly smaller in all of the tested TRPC5/TRPC1 heteromers (Figure 1G) compared to the cells overexpressing TRPC5 channels alone. This is consistent with the report by Storch et al. [33], who found that the 1:1 TRPC5/TRPC1 heteromer exhibited a low permeability to calcium. This not only further provides evidence that TRPC1 negatively regulates TRPC5 function but also indicates that increased TRPC1 expression in a cell seems to further decrease the TRPC5/TRPC1 channel ability to mediate calcium influx. This is a potential mechanism for the physiological control of TRPC5 function. Furthermore, changes in the expression of TRPC proteins have been seen in response to both internal and external stimuli. In animal models, expression changes have been seen for TRPC proteins in response to the estrous cycle or the temperature of their environment [46,47]. In humans, studies have shown increased TRPC1 expression in response to heart failure and hypertrophic cardiomyopathy [48]. This could provide the body with a method of changing the TRPC heteromer properties in response to the environment by altering the expression levels of TRPC1, TRPC5, or both. In fact, research suggests that TRPC1 and TRPC5 have opposing regulatory effects on the neurite development in a PC12 cell model, which would support our assertion that the TRPC5:TRPC1 ratio can be used to regulate cell activities through changing the heteromeric channel properties [17,49].

Kollewe et al. [50] reported that, in the rodent brain, the core of native TRPC5/TRPC1-containing channels are mostly heteromers composed of one TRPC1 subunit and three TRPC5 subunits. The authors used advanced high-resolution proteomics combining the affinity purification of native protein complexes and nano-flow liquid chromatography tandem mass spectrometry to determine the native TRPC5/TRPC1 channel stoichiometry. Our data are consistent with this conclusion and further reveal that increasing the relative number of TRPC1 in the heteromeric channel would reduce cation influx through the heteromeric TRPC5/TRPC1 channel.

In this study, we observed very small currents in the HEK cells expressing the TRPC5-TRPC1 concatemer alone (Figure 2B). This was unexpected because the same TRPC5-TRPC1 concatemer expressed in another HEK cell subline showed larger histamine-induced inward currents (Supplemental Figure S1). It is possible that the previous batch of cells had an endogenous expression of human TRPC5. Since the cells were lost, we cannot confirm its human TRPC5 expression status.

Herein, we explored the molecular determinants of TRPC1 contributing to the dose-dependent attenuation of the heteromeric TRPC5/TRPC1 channel function. Experimental evidence shows that mutating the lysine residues located above (K655) and below (K663) the putative lower gate of the heteromeric TRPC5/TRPC1 channel has a significant effect on the biophysical properties of the heteromers. The single mutations of K655N or K663N in TRPC1 individually show significantly larger TRPC5/TRPC1 heteromeric currents when compared to the WT heteromeric channels. This suggests that these residues may underlie the inhibitory effect of TRPC1 on TRPC5 function. This supports our hypothesis that the two positively charged lysine residues are affecting the cation permeability of the TRPC5/TRPC1 heteromeric ion channels.

The effect of negatively charged residues on ion channel conductance was described before. The nicotinic acetylcholine receptor channel is also composed of several subunits and has been found to have negatively charged rings in the lumen of its pore that, in part, determine the amplitude of cation currents through the heteromeric channel. Some of these subunits have more negative charges than others, and studies have shown that channels composed of subunits with more negatively charged residues show greater currents [51]. The importance of luminal positively charged amino acid residues in regulating the permeability of chloride channels was demonstrated [52]. However, the regulatory role of positively charged residues in the lumen of a TRPC channel pore has not been previously described. Lysine is positively charged at all physiological pH values. In the TRPC5/TRPC1 heteromeric channels, positively charged lysines located in the lumen of the channel’s pore would likely increase the repulsive forces on the positive cations passing through the pore, leading to a decreased current when there are more TRPC1 subunits, and therefore more positive lysines present in the channel pore.

We noted that the shape of the TRPC5/TRPC1^K655N^ mutant’s I–V was different compared with the I–V of the WT TRPC5/TRPC1 channels. As the membrane potential increased to unphysiologically high positive values, the outward TRPC5/TRPC1^K655N^ current stopped increasing and leveled off, while, in the WT and TRPC5/TRPC1^K663N^, the current kept increasing with the rising membrane potential (Figure 3). We initially hypothesized that low lysine can block the low gate of the channel while being drawn towards the extracellular end of the pore by strong negative extracellular membrane potentials during depolarizing voltage ramps. Our homology model, shown in Figure 3A,B, predicted that K655 and K663 residues are lining the channel pore lumen and flanking the low channel gate. Since these residues are both positively charged, they would repel one another. We expected that, because the substitution of the upper lysine (K655) with the asparagine would decrease the downward force of the upper lysine on the lower lysine (K663), this would allow the lower lysine to swing up and physically block the flow of cations at strong positive holding potentials. Our initial mutagenesis study with the single mutations were consistent with our hypothesis showing that substituting K655 with asparagine revealed the I–V shape change, and that substituting K663 showed an I–V curve resembling that of the wild-type heteromers (Figure 3C–E). However, our further experiments with the double mutant of K655N and K663N did not support this hypothesis, as the heteromers with the double-mutant TRPC1 still showed the plateau with greater depolarization.

Despite our initial hypothesis being incorrect, it is clear that substituting the K655 residue with asparagine has an effect on the I–V curve of the TRPC5/TRPC1 heteromeric channels. It is possible that such a substitution of the K655 lysine with asparagine might create a new intracellular site allowing, for example, a voltage-dependent Cs+-mediated open channel pore block. Indeed, voltage-dependent block by intracellular Na+ cations was reported for large conductance Ca^2+^-activated potassium channels during comparable strong depolarizations of the membrane [53]. More structural analysis studies should be performed to confirm or refute such a revised hypothesis. However, thus far, there is no crystal structure of the open TRPC5 or TRPC1 channel. Therefore, we cannot determine whether such changes occur during heteromeric channel gating.

Another important result of these experiments is the discovery of a volage-dependent block by extracellular Mg^2+^ in the TRPC5/TRPC1 heteromeric channels (Figure 4). The shape of I–V relationship of heteromeric TRPC5/TRPC1 resembled the I–V relationship of the NMDA receptor channel at physiological negative membrane potentials, which is due to a potent pore block by extracellular Mg^2+^. This is a well-studied and physiologically important phenomenon for glutamatergic signaling in the brain [54,55]. While the wild type of TRPC5/TRPC1 heteromeric channels showed a weak Mg^2+^ block, the mutated channels both showed a significantly larger Mg^2+^ block compared to the wild-type channels (Figure 4E). The residues that were mutated lay within the pore region of the heteromeric channels. Therefore, this might indicate that there is a Mg^2+^ binding site in the pore of the heteromers, which is likely close to the lower gate of the channel pore. A similar mechanism was reported to explain the voltage-dependent block of the NMDA channel by extracellular Mg^2+^, which binds within the NMDA channel pore [56,57,58]. For the TRPC5/TRPC1 channel, it is also possible that mutating the pore’s lysine residues decreased the overall positive surface charge of the pore, which decreased the electrostatic repulsion of Mg^2+^ and allowed for a more complete pore block. This property of TRPC5/TRPC1 channels could be important for regulating the channel function. More experiments will be needed to better understand the physiological meaning of the TRPC5/TRPC1 Mg^2+^ block, and how it relates to the structure and function of these heteromeric TRPC channels.

We also studied a mutant of the TRPC5/TRPC1 heteromeric channel containing TRPC1, with both lysines mutated to asparagines (the double mutant of TRPC1^K655N,K663N^). We found that the TRPC5/TRPC1^K655N,K663N^ heteromers at a 1:3 relative stoichiometry had a significantly larger current compared to the TRPC5/TRPC1 (WT) of a 1:3 relative stoichiometry, which had very little or no current. The inward currents through the double mutant of TRPC5/TRPC1 at a ratio of 1:3 were not significantly different than those through the double mutant with a higher TRPC5:TRPC1 ratio (3:1). In stark contrast, wild-type TRPC5/TRPC1 heteromers at a relative stoichiometry of 1:3 exhibited significantly smaller inward currents compared to those in wild-type TRPC5/TRPC1 heteromers with a relative stoichiometry of 3:1 (Figure 1). Thus, replacing both positively charged residues with asparagines in the TRPC1 subunit of the TRPC5/TRPC1 heteromeric channel shows an attenuation of the inhibitory effect of TRPC1 on the TRPC5 function at physiologically negative potentials. These data reveal a potential mechanism of the negative regulation of TRPC5 activity by TRPC1. However, the change does not seem to be additive, as currents through the double-mutant TRPC5/TRPC1^K63N,K655N^ heteromeric channels did not show significantly greater current than either of the single-mutant heteromeric channels.

Our study has several limitations. We did not assess whether the indicated mutations affected the pharmacological properties of the heteromeric TRPC5/TRPC1 channels. Thus, future experiments will be needed to determine whether the lysine substitutions alter the ability of known modulators of TRPC5/TRPC1 heteromers to affect its function. During this study, we estimated the permeability ratios to Ca^2+^ and Na^+^ for TRPC5 and heteromeric TRPC5/TRPC1 channels with variable relative subunit expression to confirm the observation of Storch et al. [33] that the heteromeric TRPC5/TRPC1 channel exhibits a reduced calcium permeability compared to that of TRPC5. Although our results are consistent with the data reported by Storch et al., we noticed a large variability in our data sets, which may be due to the fact that some current amplitudes were very small, complicating the precise reversal potential determination. Furthermore, the values of the reversal potentials were not corrected for the liquid junction potential. Thus, these permeability ratio results should be interpreted with caution, although statistical significance was detected. Additionally, since it is known that TRPC1 can be retained in the endoplasmic reticulum in HEK cells, we cannot rule out that the increased expression of TRPC1 can hinder the plasma membrane trafficking of TRPC5-TRPC1 concatemers or the TRPC5 protein by obstructing their endoplasmic reticulum exit. We qualitatively demonstrated that the heteromers with a relative stoichiometry of 1:3 for TRPC5/TRPC1, which shows little current, are present in the plasma membrane (Supplemental Figure S2). However, further quantitative biochemical experiments will be needed to determine whether TRPC5 or TRPC5-TRPC1 trafficking is affected when TRPC1 expression levels are increased in cells. We also realize that changes in the expression level of a TRPC subunit during transfection may not necessarily result in a precisely reciprocal alteration in the TRPC5/TRPC1 subunit stoichiometry. Furthermore, in the case of the TRPC5-TRPC1 concatemers co-assembled alone or with either TRPC1 or TRPC5 monomeric subunits, we cannot exclude the possibility of the formation of some heteromers with variable pore structures, as was shown for other ion channels [59,60], where only a part of the concatemer was incorporated into the resulting heteromeric channels. However, evidence exists that heteromeric channels may predominantly incorporate the entire concatenated dimer, at least in cyclic nucleotide-gated tetrameric channels [61]. Remarkably, natural concatemers are critical for the formation of the native weak inwardly rectifying two-pore domain K^+^ TwiK channel [62].

## 5. Conclusions

In this work, we present the first evidence that TRPC1 negatively regulates TRPC5 activity via a mechanism involving positively charged lysines facing the lumen of the heteromeric channel pore. We showed that inward currents through the heteromeric TRPC5/TRPC1 channel increase with decreased TRPC1 expression, favoring a relative stoichiometry of 1:3 for TRPC1/TRPC5 heteromers. We show that the substitution of positively charged luminal lysine residues with asparagine increased the total current through TRPC5/TRPC1 heteromers. This suggests that these specific lysine residues may be involved in the negative regulatory role that TRPC1 has over the TRPC5 protein. We also revealed that the heteromeric TRPC5/TRPC1 channel function may be regulated via an extracellular Mg^2+^ voltage-dependent block resembling that of the NMDA receptor channel block by extracellular Mg^2+^. These data indicate that there may be a unique physiological mechanism regulating TRPC5 channel function by the variable degree of heteromerization with TRPC1 subunits. Our data are consistent with the results reported by Kollewe et al. [50], indicating that the native TRPC1/TRPC5 channel in the rodent brain exhibits a defined stoichiometry of 1:3. Our new findings may lead to a better understanding of how heteromeric TRPC5/TRPC1 channel biophysical properties are regulated. Novel knowledge in this area of research could be a key to better treatment for neurological and some vascular diseases associated with increased TRPC5 activity.

## Data Availability

All data are included herein.

## Supplementary Materials

The following supporting information can be downloaded at: https://www.mdpi.com/article/10.3390/cells13232019/s1, Figure S1: Expression of TRPC5-TRPC1 concatemers alone or in combination with either TRPC5 or TRPC1 in an older high-passage HEK cell line. A–C show averaged peak current density-voltage relationships. D. Comparison of peak current densities determined at −60 and +100 mV in the tested groups shown in A–C; Figure S2: Qualitative biotinylation experiments confirmed that TRPC5-TRPC1 concatemers were present in the plasma membrane. Two separate immunoblots are shown. The blots were probed with the primary monoclonal anti-TRPC1 antibodies (a gift from Tsiokas lab). “5” indicates the lysates from biotinylated HEK cells expressing TRPC5 alone. “51” indicates the lysates from biotinylated HEK cells expressing TRPC5-TRPC1 tandems. “515” indicates the lysates from biotinylated HEK cells co-expressing TRPC5-TRPC1 tandems and TRPC5. “511” indicates the lysates from biotinylated HEK cells co-expressing TRPC5-TRPC1 tandems and TRPC1. “H” indicates the groups that were treated with 10 μM histamine before the biotinylation procedure.
