# Supplementary material for: Effects of TRPC1’s Lysines on Heteromeric TRPC5-TRPC1 Channel Function
Source: Cells. 2024 Dec 6;13(23):2019. doi: 10.3390/cells13232019 (PMC11640535; doi:10.3390/cells13232019)
Supplement: Supplementary file 1 [file cells-13-02019-s001.zip › cells-3295563-supplementary.pdf]

Supplemental Figures

Article

Effects of TRPC1's Lysines on Heteromeric TRPC5-TRPC1 Channel Function

Isaac S. Demaree [1], Sanjay Kumar [1,2], Kayla Tennessen [3], Quyen Q. Hoang [3,4], Fletcher A. White [4,5] and Alexander G. Obukhov [1,4,*]

1 Department of Anatomy, Cell Biology & Physiology, Indiana University School of Medicine, Indianapolis, IN 46202, USA; idemaree@iu.edu (I.S.D.); sanjaycdri@gmail.com (S.K.)
2 Department of Life Sciences, School of Earth, Biological, and Environmental Sciences, Central University of South Bihar, Gaya 824236, India
3 Department of Biochemistry and Molecular Biology, Indiana University School of Medicine, Indianapolis, IN 46202, USA; ktenness@iu.edu (K.T.); qqhoang@iu.edu (Q.Q.H.)
4 Stark Neurosciences Research Institute, Indiana University School of Medicine, Indianapolis, IN 46202, USA; fawhite@iu.edu
5 Department of Anesthesia, Indiana University School of Medicine, Indianapolis, IN 46202, USA
* Correspondence: aobukhov@iu.edu; Tel.: +1-317-274-8078

Supplemental Figure S1

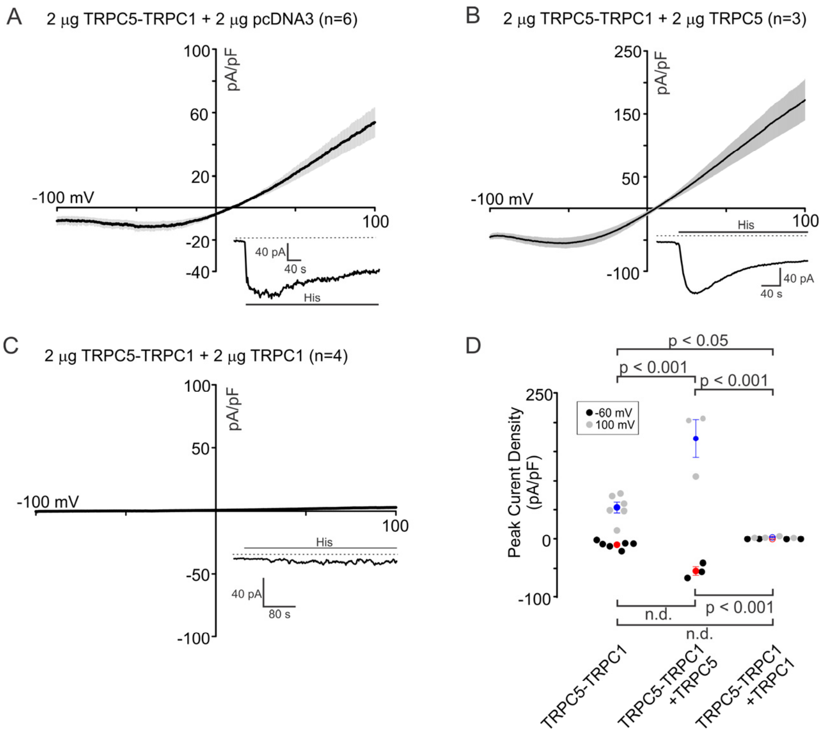

Supplemental Figure S1. Expression of TRPC5-TRPC1 concatemers alone or in combination with either TRPC5 or TRPC1 in an older high-passage HEK cell line. **A-C** show averaged peak current density-voltage relationships. **D.** Comparison of peak current densities determined at -60 and +100 mV in the tested groups shown in **A-C**.

Supplemental Figure S2

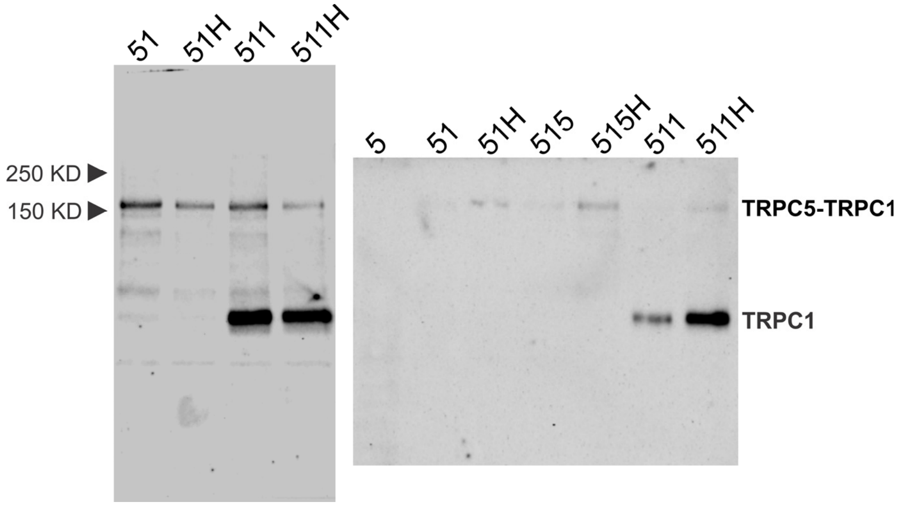

Supplemental Figure S2. Qualitative biotinylation experiments confirmed that TRPC5-TRPC1 concatemers were present in the plasma membrane. Two separate immunoblots are shown. The blots were probed with the primary monoclonal anti-TRPC1 antibodies (a gift from Tsiokas lab). "5" indicates the lysates from biotinylated HEK cells expressing TRPC5 alone. "51" indicates the lysates from biotinylated HEK cells expressing TRPC5-TRPC1 tandems. "515" indicates the lysates from biotinylated HEK cells co-expressing TRPC5-TRPC1 tandems and TRPC5. "511" indicates the lysates from biotinylated HEK cells co-expressing TRPC5-TRPC1 tandems and TRPC1. "H" indicates the groups that were treated with 10 μM histamine before the biotinylation procedure.
